# A computer-assisted motivational social network intervention to reduce alcohol, drug and HIV risk behaviors among Housing First residents

**DOI:** 10.1186/s13722-016-0052-y

**Published:** 2016-03-15

**Authors:** David P. Kennedy, Sarah B. Hunter, Karen Chan Osilla, Ervant Maksabedian, Daniela Golinelli, Joan S. Tucker

**Affiliations:** RAND, 1776 Main Street, Santa Monica, CA 90407-2138 USA

**Keywords:** Social network intervention, HIV risk behaviors, Data visualization, Alcohol and other drug use, Homelessness, Housing First, Motivational interviewing, EgoWeb

## Abstract

**Background:**

Individuals transitioning from homelessness to housing face challenges to reducing alcohol, drug and HIV risk behaviors. To aid in this transition, this study developed and will test a computer-assisted intervention that delivers personalized social network feedback by an intervention facilitator trained in motivational interviewing (MI). The intervention goal is to enhance motivation to reduce high risk alcohol and other drug (AOD) use and reduce HIV risk behaviors.

**Methods/design:**

In this Stage 1b pilot trial, 60 individuals that are transitioning from homelessness to housing will be randomly assigned to the intervention or control condition. The intervention condition consists of four biweekly social network sessions conducted using MI. AOD use and HIV risk behaviors will be monitored prior to and immediately following the intervention and compared to control participants’ behaviors to explore whether the intervention was associated with any systematic changes in AOD use or HIV risk behaviors.

**Discussion:**

Social network health interventions are an innovative approach for reducing future AOD use and HIV risk problems, but little is known about their feasibility, acceptability, and efficacy. The current study develops and pilot-tests a computer-assisted intervention that incorporates social network visualizations and MI techniques to reduce high risk AOD use and HIV behaviors among the formerly homeless.

**ClinicalTrials.gov Identifier:**

NCT02140359.

## Background

Homeless individuals have significantly higher rates of morbidity and mortality as compared to those living in stable housing [1–4]. Among the most pressing health issues facing homeless individuals are the interrelated problems of alcohol and other drug (AOD) use and HIV risk. An estimated 30–50 % of homeless individuals are AOD dependent [5, 6]. AOD use is both a leading cause of homelessness [5] and is exacerbated by the stress of life on the street and exposure to high rates of AOD use among other homeless people [6–8]. Exposure to HIV is a significant health threat for homeless individuals, with rates of HIV infection being three to nine times greater among homeless persons than those with stable housing [4, 9, 10]. The greater vulnerability to HIV among homeless individuals is due, in part, to AOD use increasing their likelihood of engaging in risky sexual behaviors such as having unprotected sex and trading sex.

Given the association between homelessness and poorer health, the provision of stable housing is often seen as an important health intervention [11]. There are two main approaches to housing provision to homeless individuals: programs that make housing contingent on AOD abstinence and treatment participation (i.e., “treatment first” (TF) approaches [12–14]) and programs that provide housing without the abstinence requirement, known as Housing First (HF) approaches [15–17]. Studies have demonstrated that HF residents have similar [18] or improved [17, 19] AOD outcomes after 1–2 years compared to TF residents. Further, HF participants have a 53 % reduction in health service expenses compared to those on waiting lists [20]. Due to these encouraging findings, HF programs have flourished over the past two decades, there are now 100s of HF programs located across the United States [15, 17, 21, 22], and HF programs are now being developed and tested outside of the United States [23].

Although there is promise that HF programs can successfully provide housing without requiring AOD use abstinence, there are still concerns that HF programs are not necessarily a panacea to homelessness [15] and that these programs may be more helpful for those with less severe AOD use [12]. One concern raised is that individuals attempting to abstain from AOD use are inadvertently exposed to high-risk behavior because of the policy of not requiring abstinence in the environment in which the resident is living [15]. This may be particularly challenging in project-based HF models where individuals reside nearby others engaging in high risk behaviors. In these programs, HF residents may require support to face the challenge of entering a new social environment that includes a mixture of AOD users and non-users.

### Social networks and health interventions

A social network based intervention [24] may be a promising approach to enhance the positive behavioral outcomes associated with these programs. Social networks are naturally occurring groups of people which can be characterized in terms of their composition (defined as the quantity and type of individuals in a network) and social network structure (defined as the connections among network members) [25]. Social networks have the potential to influence health behaviors and outcomes through social comparison, social sanctions and rewards, information transmission, support and resources, stress reduction, and socialization [24–29]. A large and growing body of research demonstrates both the positive and negative influences of social network on the lives of homeless individuals [30–32], including their health and health-related behaviors [7, 9, 33, 34]. For example, social networks are an important source of social support and thus may influence (positively or negatively) AOD use, HIV risk behavior, AOD treatment program participation, and successful transition out of homelessness [6–8, 17, 33–39]. The social networks of homeless individuals tend to be compositionally diverse, often including non-homeless individuals who influence AOD behavior [7, 40–43] and a mixture of risky and non-risky influences and sources of potential social support [7, 44]. These networks tend to be structurally diverse as well [44, 45], with a range of interconnections among high-risk and/or supportive network contacts [46].

Given these findings, targeting the social networks of HF residents may be a promising approach to support the transition from homelessness and reduce AOD- and HIV-related risk behaviors. Social network based interventions have shown promise in changing health behaviors [24]. For example, studies have successfully changed substance use behavior of adolescents through use of social network analysis to identify highly central peers to lead the interventions [47, 48]. Other experimental studies that have manipulated social network exposures have produced evidence of changes in behavior related to seeking health information through peer influence [49]. However, there are limitations to incorporating existing social network intervention approaches into HF programs. Most existing network-based AOD interventions primarily target the composition of networks. For example, many are based on abstinence and/or focus on developing new social networks with other abstaining network contacts [50–56]. These interventions are only applicable to those who are already abstaining from AOD use. Few social network based interventions target changes to network structure [57]. Those that account for network structure are primarily educational interventions that use social network analysis to track the impact of the intervention throughout the network [24]. In this approach, key individuals in the group are selected to receive an intervention because of their central network position and greater likelihood of spreading the intervention to their peers. This approach would not address the diversity of network contacts for homeless people or the shifts in network composition and structure they are likely to experience as they transition into HF programs. New HF residents are likely to face a reduction in their contact with homeless peers, increased contact with persons who reside close to them, and possibly reconnect with network members from their past.

### Personal network visualizations

This study involves designing a personalized social network intervention that targets behavioral change through personal network visualization presentations by a facilitator trained in motivational interviewing (MI) [45]. We hypothesize that a social network-based intervention that focuses on HF residents’ *personal networks* could assist individuals in making positive compositional changes (i.e., adding and/or removing types of network members) and structural changes (i.e., strengthening, weakening, adding connections among network members) to their networks that are associated with reduced AOD use and HIV sexual risk behaviors. Providing feedback on personal network composition and structure may be useful for individuals transitioning to supportive housing. As they begin to make a major shift in their physical surroundings, they can better understand their current social environment (who they are currently connected to, what types of behaviors they engage in, how these people are connected to each other) to help guide their interactions with these people in the future. To date, we are unaware of any social network interventions that target the diversity of social contacts and social structure experienced by people as they transition from homelessness.

Personal network interviews can be enhanced with visualizations of the networks to trigger additional discussion about the network dynamics of a respondent [45, 58–60]. Personal network visualizations allow for display of both compositional and structural features of the network. For example, interview participants can see who within their networks is engaging in risky behavior, providing them with support, and how these different types of individuals are connected to each other. These visualizations are easy to understand and have been used with homeless participants with less than high school education [41, 45]. Although it is often assumed that the presentation of network visualizations can lead to behavior change [24], to our knowledge these types of visualizations have not been incorporated into AOD use and HIV risk reduction interventions. Network visualizations are not new to health-related interventions. Visualization tools have been used by social workers to provide feedback to families about health and social support [61, 62]. However, these techniques only provide information about network composition and do not incorporate structure. Network visualizations that incorporate structure have been used in a study of an educational intervention that promoted interactions among adolescents [63]. This study found that presentation of network visualizations prompted students to become more strategic about how they formed connections to other students to improve their learning performance. We hypothesize that HF residents could be impacted in a similar way.

### Computer assisted motivational interviewing

Presenting visual personalized social network feedback to participants using MI may be an ideal approach to help participants explore changes to their social network and risk behaviors. MI is an evidence-based intervention style [64] that has been used to reduce AOD abuse and HIV risk behavior [57]. More specifically, MI is a client-centered, directive therapeutic approach to enhance readiness for change by helping individuals explore and resolve ambivalence [65]. Adapted from self-perception theory that posits that people become more committed to a position that they hear themselves state [66], MI seeks to elicit “change talk”—individual expressions of desire, ability, reasons, and need for change. MI facilitators are instructed to respond using “reflective listening”, which is to offer periodic summaries of an individual’s self-motivational statements [67]. As a result, individuals hear themselves explain their own motivations for change, and also hear them reflected back again by a MI facilitator thereby increasing desire and commitment to changing a behavior. Recently, one existing MI intervention has been enhanced with a social network component [57]. However, currently, there are no MI interventions to our knowledge that take into account both compositional and structural characteristics of social networks with visualizations and none that target HF residents.

Developing a computer-assisted tool for delivering a social network intervention may provide a cost-effective way of disseminating it. Computer-assisted intervention delivery tools offer low cost options for adoption in community settings and improve fidelity to intervention delivery procedures [68] using a variety of computer sources such as laptops and tablets. Use of a tool to collect and present personal network information can also allow individuals to visualize how their personal networks change over time, which may provide tangible evidence that the steps they are taking to make changes are having an effect, reinforcing the intervention’s benefits.

### Study design, aims and hypotheses

This study was designed to explore whether employing a MI approach to present personal network information electronically would help HF residents transition away from high-risk social networks and toward low-risk, supportive social networks and result in less risky AOD use and sexual behaviors. Consistent with the stage model approach to behavioral therapy intervention development [69], the study consists of two phases, a development phase (i.e., Stage 1a) and a pilot test phase (i.e., Stage 1b). The Stage 1a development work has been completed and consisted of developing and iteratively testing the intervention to assess usability, feasibility and acceptability among residential support services staff and HF residents. Data analysis of qualitative data generated during Stage 1a is ongoing and will be reported elsewhere. This paper describes the design of the Stage 1b pilot test of the intervention.

The aim of the pilot study is to generate preliminary evidence about the effectiveness of the intervention on reducing AOD use and HIV risk behaviors. To meet this aim, 60 residents with past year harmful AOD use will be randomly assigned to receive the intervention or usual care. The justification of a sample size of 30 per study condition is based on published guidelines for conducting Stage 1b clinical trials which are considered pilot studies, developmental in scope, and exploratory in nature [69]. We will analyze baseline (i.e., pre-intervention) and follow-up (i.e., post-intervention, approximately 90 days from baseline) data to determine the feasibility, acceptability and promise of the intervention for a subsequent Stage 2 efficacy trial [69].

We will explore the following hypotheses: intervention participants, compared to control participants, will show: (a) increased AOD use and safe sex-related self-efficacy (b) increased readiness to change AOD use and high-risk sexual behavior, (c) social networks with fewer and less central high-risk members at the follow-up assessment and (d) significant reductions in AOD use and fewer HIV sexual risk behaviors between the baseline and follow-up period compared to control participants. We examine self-efficacy and readiness to change as those appear to be the psychological constructs that MI is designed to address. We hypothesize that the motivational social network intervention will increase self-efficacy and readiness to reduce AOD use and HIV risk behaviors. We will also explore whether the proximal outcomes (i.e., greater efficacy, greater readiness to change, and reductions in the riskiness of networks) appear to mediate the relationship between the intervention and the distal outcomes (i.e., changes in high-risk behavior). At the end of this study, we will have an electronic tool for delivering a motivational network intervention (MNI), a well-described set of procedures for conducting the MNI intervention, and preliminary data for a possible future efficacy trial.

## Methods

### Setting

The study is being conducted in collaboration with Skid Row Housing Trust (SRHT) and Single Room Occupancy Housing Corporation (SRO Housing), two of the largest HF providers in Los Angeles County. Both organizations combine the HF framework for providing rapid housing with minimal preconditions with permanent supportive housing (PSH) to many of its residents [70]. PSH is long-term community-based housing linked with voluntary supportive services for individuals with disabilities who have experienced homelessness. SRHT and SRO Housing manage properties in the Skid Row area of downtown Los Angeles, which has the largest concentrations of homeless adults in Los Angeles County.

### Participants, eligibility and recruitment

**Fig. 1:**
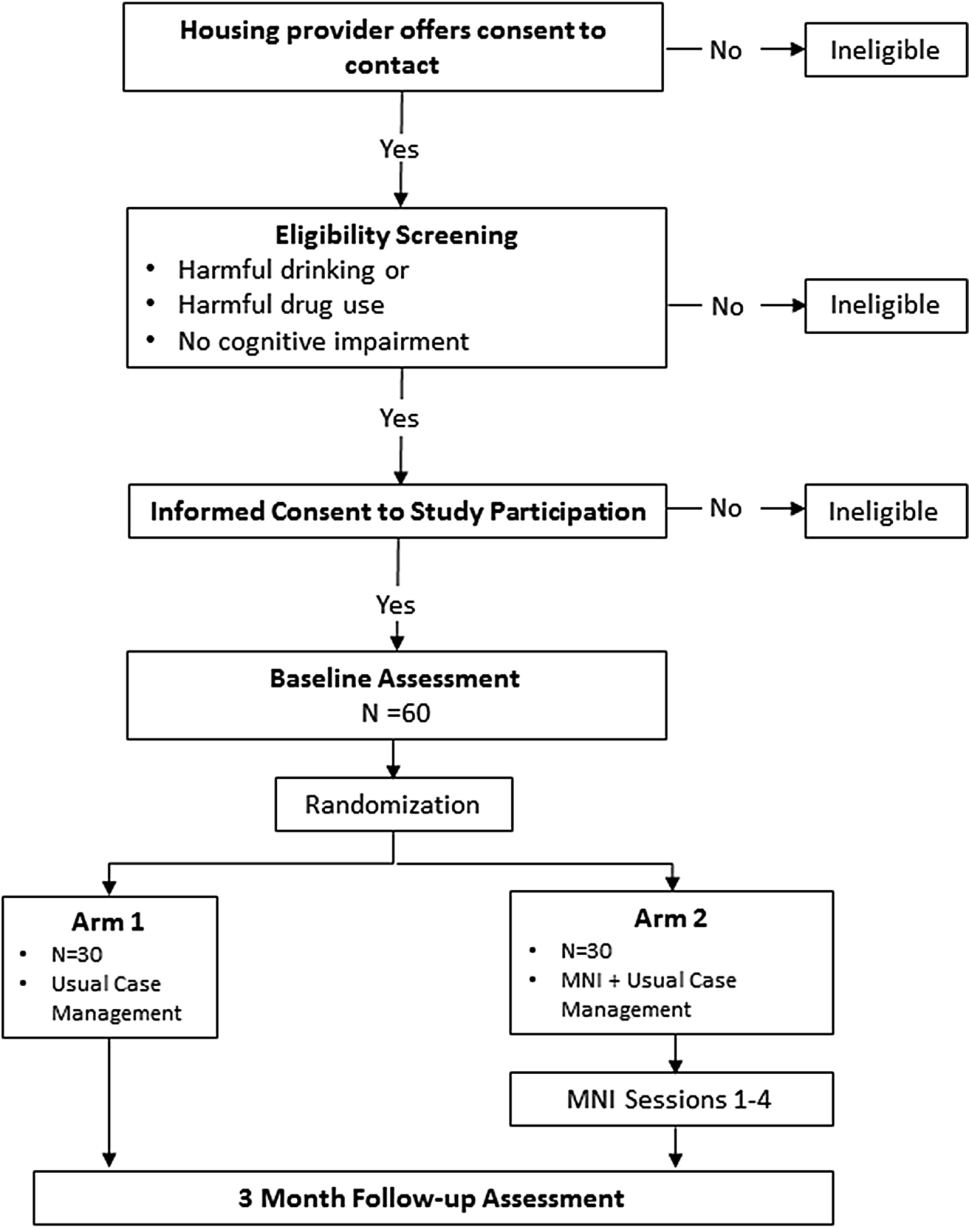
Study design and procedures

Participants will include recently housed residents aged 18 or older who are (1) able to speak and understand English, (2) are not cognitively impaired (based on administration of the Short Blessed Scale Exam during screening for eligibility [71]), and (3) screen positive for past-year harmful AOD use (either drugs or alcohol) using the Alcohol Use Disorders Identification Test (AUDIT-C) (a score >4 for men and >3 for women) [72] and the Drug Abuse Screen Test (DAST) (a score >2) [73–75]. Recruitment, screening for eligibility, and assessment procedures are depicted in Fig. 1.

Leasing staff will be instructed to introduce the study to all housing applicants that have recently received assignment to a housing unit. Those who express interest in being contacted for the study will be given a consent-to-be-contacted form to complete. Leasing office staff will share the completed consent-to-be-contacted form with the research team. Next, the research team will attempt to contact the potential participant by phone to complete a study screener. If a resident is interested in the study but does not have access to their own phone, the leasing staff will provide access to a phone. If the resident does not contact research staff by phone at leasing and does not provide a working phone number on the consent-to-be-contacted form, a member of the research team will attempt to locate the resident at their residence and provide access to a phone to complete the study screener. Once an interested resident is connected by phone for screening, the research team will describe the study, obtain consent to conduct the screener, and conduct the screener interview to determine eligibility. Next, eligible participants will be asked to schedule an in-person study consent and baseline assessment. Participants will also be contacted for a follow-up assessment 3 months after baseline. Residents will be provided with study appointment cards to assist them in recalling their appointments. Participants will be paid $30 for the baseline (i.e., pre-intervention) assessment and $40 for the follow-up (i.e., post-intervention, approximately 90 days after baseline) assessment.

### Randomization

After participants complete the baseline assessment, they will be assigned to one of two experimental conditions (i.e., intervention or control). We aim to randomly assign 60 participants using a stratified permuted block randomization strategy. Given the relatively small sample size, the randomization might produce an unbalanced assignment with respect to gender. For this reason the randomization will be stratified. About 26 % of SRHT clients are women and stratifying on this characteristic will ensure an approximately equal number of women assigned to both conditions. If the intervention and control groups turn out to be unbalanced with respect to other characteristics, we will use covariate adjustment at the analysis phase. Next, participants assigned to the intervention arm will be contacted by project staff by phone to schedule the four MNI sessions, one every 2 weeks. If the participant is difficult to reach by phone, a project staff member will attempt to contact the participant by visiting their residence to schedule their first session. Participants will be provided with appointment cards as reminders. Participants will be given a $5 food gift card for each intervention session they attend.

### MNI intervention description

**Fig. 2:**
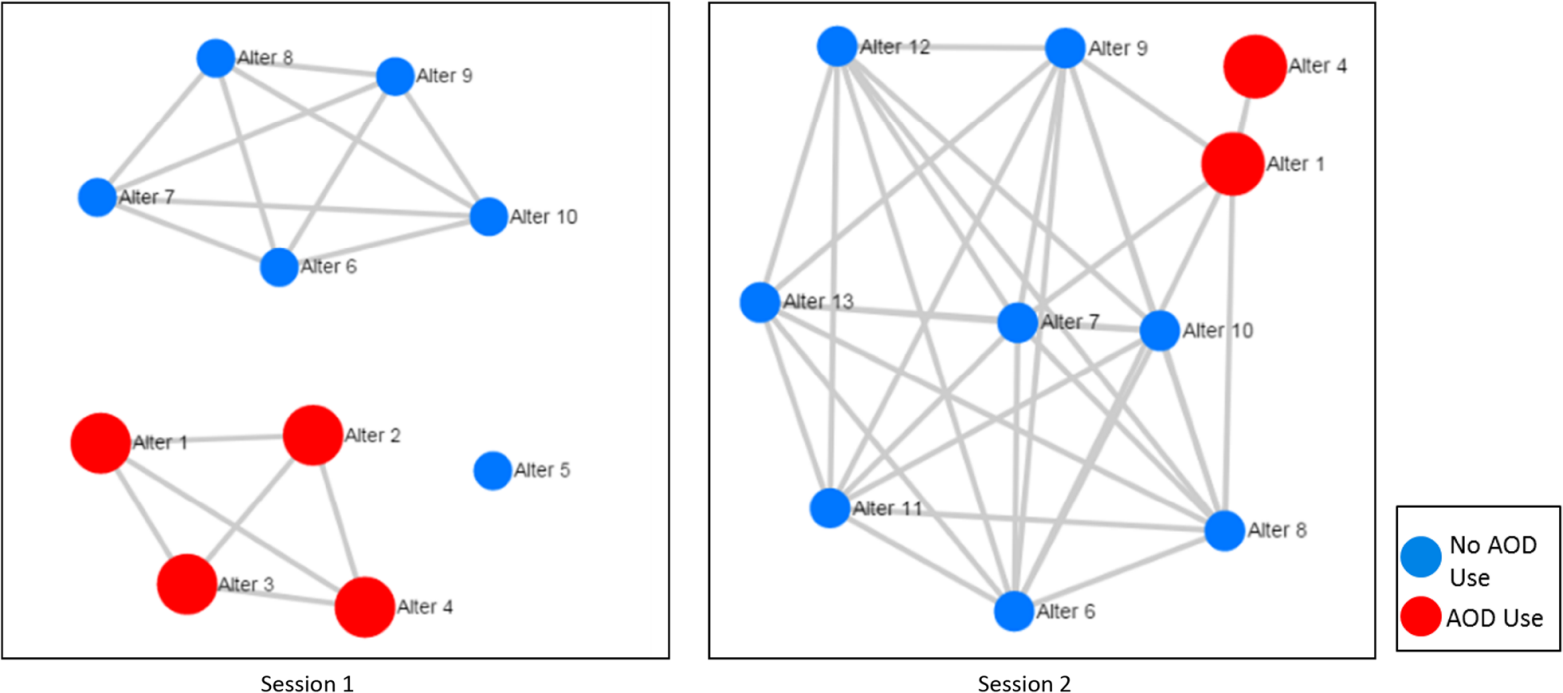
Hypothetical MNI participant network visualizations across two sessions. *Each diagram* demonstrates network members (“alters”) with *circles* (“nodes”) and *lines* (“edges”) connecting alters who have interacted with each other in the past 2 weeks. The *left* hand diagram (Session 1) depicts a network of ten nodes with two separate groups (“components”) and one network member who had no interactions with other alters (an “isolate”). One component in Session 1 is comprised of all AOD users and the other includes no AOD users. The *right* hand diagram shows that three of session 1 network members are no longer part of the network and three new members have been added to the network

The MNI intervention consists of four biweekly sessions that last approximately 45–60 min and consist of two parts: a network interview with closed-ended network questions and a discussion of resulting network visualizations conducted using a MI approach. Structured network interview questions will be similar to previous studies with homeless populations [6–8, 41, 43, 76, 77]. Participants will be asked to identify people they have had contact with in the past 2 weeks, with follow-up questions about each of these contacts (e.g., likelihood that they will drink alcohol or use drugs in the next 2 weeks, if the participant drank more alcohol or used more drugs than they wanted to when with the contact; if the participant has had unprotected sex with any of the contacts in the past 2 weeks; whether any of the contacts provide social support to the participant; whether the contacts interacted in the past 2 weeks). As the participant talks, the facilitator will enter the participant’s interview responses into the computer-assisted interface, which will subsequently generate several social network visualizations based on the participant’s responses. See Fig. 2 for examples of network visualizations for a hypothetical MNI participant. These visualizations are intended to provide content for discussions during the MNI. The facilitator would show the diagram and then use MI techniques (e.g., asking evocative open-ended questions, stating reflections, elaborating on change talk) to discuss what patterns the participant notices in the diagram (e.g., how certain contacts may trigger their AOD use and how they feel about those contacts). For the example shown in Fig. 2, in Session 1, the facilitator would gather information by stating “Tell me about the different groups of people you see” and “The red circles are people you said you tend to drink more with, tell me about those people.” The facilitator would use MI techniques to explore and resolve any ambivalence about changing their AOD and risky sexual behaviors.

The second–fourth sessions are designed to build on previous discussions. Facilitators will start these sessions with a discussion of the goals identified in the previous session by asking, “Tell me how those goals have been going for you. Have things changed? Did anything get in the way?” After discussion of the goals, the session will include all of the same questions about the participant’s network they were asked in the previous sessions, including a discussion of the current and previous visualizations. For the example shown in Fig. 2, in Session 2, the facilitator might ask “What differences do you see between this week’s diagram and our first one?” The facilitator would highlight any change talk about the new people in their network who do not use AOD (i.e., changes in network structure) and the strategies they used to change their network composition (e.g., fewer who are using AOD). The facilitator would also explore whether the participant is willing to interact less with the two remaining people who do use AOD in their network.

This four session strategy will allow time for network change strategies to be put into place and for participants to discuss change experiences with the intervention facilitators. For example, participants may discuss wanting to spend more or less time with network contacts, wanting to connect network members who do not know each other, or ties to network contacts that they would like to break. Participants may not be able to put strategies to achieve these goals in place right away or they may need to try different strategies. Initial changes in network structure or composition may trigger the opportunity to develop strategies for secondary or tertiary changes. Also, participants may require encouragement from facilitators to maintain initial strategies. Four sessions will allow ongoing discussions of this process. Also, some of their strategies may produce unexpected negative results and they will be able to discuss these unexpected effects with facilitators who can help them develop alternative strategies. In summary, the visualizations resulting from the network interview are designed to provide both intervention facilitators and participants with a tangible object to focus questions and answers and make abstract discussions of social life, relationships, and social change more concrete.

### Usual care

Participants assigned to both the intervention and control conditions will receive usual case management support provided by SRHT and SRO Housing. After an initial case management intake assessment that is scheduled to occur within the first 30 days that a resident is housed, residents are expected to meet with case managers regularly for up to 60–90 days, based on need. Case management meetings last 15–60 min and are customized to the residents’ needs. Case managers facilitate residents’ access to on-site services, such as support groups, as well as make referrals to offsite services.

### Intervention fidelity monitoring and measurement

Intervention facilitators will be trained in MI techniques and a clinical psychologist affiliated with the motivational interviewing network of trainers (MINT) will review the recording of each session to provide regular feedback and re-training, as necessary. Fidelity to the intervention protocol will be assessed with an 8-item scale to determine if key components of the intervention were discussed, similar to previous studies [78–80]. The fidelity checklist will consist of a five-point Likert scale with response options ranging from “completely”, “partially”, to “not at all” covered. For example, items will assess whether the facilitator discussed the client’s desires to make network changes, such as interacting with specific network ties more or less frequently.

### Measures

Table 1 gives an overview of the pilot study measures and the phase in which they will be assessed. Study measures were selected based on their use in similar populations, including low income, low education AOD using populations [81, 82] and populations engaging in heavy AOD use and high-risk sexual behavior [83]. Many of the measures have also been recommended by the VA Mental Health QUERI taskforce [84] and NIDA [85]. Because of the pilot nature of this study, only one follow-up assessment will be conducted. We plan to assess participants within 2 weeks after the final MNI session in order to measure the immediate benefits of the intervention rather than longer-term effects. The assessments will be collected in a one-on-one in-person interview by an independent data collection organization using EgoWeb as a computer-assisted personal interviewing (CAPI) software. The data collectors will be blind to the study condition assigned to the participant.

**Table 1:** Study measures and administrative timeline

	Screening	Baseline	Follow-up
At-risk drinking: AUDIT-C	X		
Any illegal drug use: DAST	X		
Demographics: age, gender, race/ethnicity, education		X	
Lifetime experience with homelessness		X	
Recent experience with homelessness		X	X
Readiness to change: RTCQ, RTCQ-SB, contemplation ladders		X	X
Self-efficacy: AASE, condom efficacy		X	X
AOD use: Quantity Frequency Index (QFI)		X	X
High-risk sex: unprotected sex, concurrent partnerships		X	X
Social network: composition and structure of drinking/drug/abstinent supportive network members		X	X
Client satisfaction (MNI participants only)			X

### Baseline measures

We will assess baseline *demographic characteristics* (i.e., age, gender, race/ethnicity, education, number of children, marital status, income) and history with homelessness to describe the study population, explore whether there are any differences between the participants assigned to the different study conditions, and potentially explore as potential intervention effect moderators.

### Outcome measures

We will measure both proximal and distal outcomes. Proximal outcomes are readiness and self-efficacy to change AOD use and safe-sex behavior, and social network composition and structure. Distal outcomes are AOD use behaviors and HIV risk behaviors. For the proximal outcomes, we will measure readiness to change in two ways. The first method is a one-item *contemplation ladder* adapted from the smoking literature [86] that displays a picture of a ladder with corresponding stages of change. For the substance that the participant considers their biggest current problem, they are asked to identify where they are in changing their use of the substance on a 0–10 scale (e.g., 0 = “No thought of quitting”, 5 = “Think I should quit but not quite ready”, 10 = “Taking action to quit”). A separate ladder will ask participants to give a similar rating of their willingness to change their unprotected sex behavior. The ratings that these ladders produce have adequate reliability and good validity [86] and have been used for smokers with schizophrenia [87], drinking adults with multiple sex partners [83], and welfare applicants [81]. We will also measure readiness to change using the Readiness to Change Questionnaire (RTCQ) [88–90], which consists of 12 items that assess the readiness to change AOD use (precontemplation, contemplation, preparation, or action) and the Readiness to Change Risky Sexual Behavior (RTCQ-SB) scale, which consists of 11 items adapted from the RTCQ for safe-sex behavior [83]. We will measure self-efficacy using an abbreviated version of the Alcohol Abstinence Self-Efficacy Scale (AASE), [91] which consists of five items indicating how confident participants feel in their ability to abstain when depressed, relaxed, craving substances, and offered alcohol. We will measure *self*-*efficacy to use condoms* with an instrument that has shown good reliability and concurrent validity [92] and has predicted unprotected sex in homeless populations [41, 43, 93].

We will collect *social network* data similar to previous homeless studies to measure change in network composition and structure [6–8, 43, 94–96]. Participants will be asked to name 20 network contacts (“alters”), who are at least 18 years of age, during the baseline and follow-up assessments because a minimum of 20 alters is necessary to produce unbiased estimates of network structure [97, 98] and to measure network composition [7]. Participants will be asked questions about the demographics of each of these network alters (gender, recent and lifetime homelessness), the drinking/drug use/safe-sex behavior of each alter, their relationships with each alter (emotional closeness, frequency of contact), and the relationships between each pair of alters [e.g., “Does (Alter 1) know (Alter 2)?”]. Based on aggregating questions about the social support provided by each alter, we will construct measures that are similar to measures of *Perceived Social Support* [36, 99] (i.e., the number and/or proportion of network members who provide emotional support, advice, tangible support) and measures that result from the *Important People Drug and Alcohol interview*, in which network members are rated on relationship characteristics (e.g., emotional closeness), drinking, drug use, safe-sex behavior, and their influence on the participants’ own high-risk behavior [100].

For the distal outcomes, we will use self-reported AOD use as our primary AOD use measure. At each assessment we will assess alcohol drinking frequency (never, once a month, 2–4 times a month, 2–3 times a week, 4–5 times a week, 6 or more times a week) and drinks per drinking day (typical number of drinks when drinking). For drug use, we will assess frequency of past 30 day drug use (none, once, twice, three times, once a week, twice a week, 3–4 times a week, 5–6 times a week, every day) for each of the following: marijuana, cocaine, crack, heroin, amphetamines/methamphetamines, injection drugs, drugs typically requiring a prescription “on their own”, and any other drug to get high or feel good. Frequency and intensity of use are thought to represent independent dimensions of AOD use behavior and tend to be relatively uncorrelated, making them a good choice as outcome measures [101]. We will derive a Quantity Frequency Index (QFI), which has a history of reliability and validity across various populations [102].

To measure *HIV risk behaviors*, we will ask about the respondent’s sexual behavior in the past 30-days overall and with particular partners named as alters. We will ask them how many times they had sex (vaginal or anal) and how often they used condoms when they had sex. We will also ask if they were engaging in sex with other partners concurrently. We have used these measures in studies of HIV risk among homeless women [43], youth [41], and men [93, 103].

To measure network changes, we will construct measures of how central various network members are using standard social network centrality measures [104, 105] similar to other studies of the social networks of homeless populations [6–8, 41, 43, 76, 77]. For example, we will measure *degree centrality* for each network member which is a count of the number of people connected to that network member in the network. We will also measure centrality with other measures that have been found to be associated with unprotected sex in homeless populations, such as *betweenness centrality* (a continuous measure of how often an alter lies on the shortest path between pairs of other alters in the network) [40] and *closeness centrality* (a continuous measure of how directly or indirectly connected a particular alter is to all the other members of a network) [77]. We will also calculate measures of network composition and size to determine if the intervention is associated with changes in the types and quantities of people in the network. For example, we will calculate proportion of alters who are drug users, who are supportive, who are perceived to engage in risky sex, etc. We will also calculate measures of overall structural characteristics to determine if the intervention affected overall interaction among network members. For example, we will calculate *density* at both time points. Density is an index that represents the proportion of ties that exist in a network relative to the total number of possible ties, and varies from 0 to 1. We will also calculate *centralization*, which is another index between 0 and 1 that measures the degree to which one or a few individuals in the network maintain the majority of ties.

During the post-intervention assessment, MNI participants will answer 23 questions about the quality of and their satisfaction with the MNI sessions they attended on a five-point Likert scale, with a higher score representing higher quality and satisfaction (e.g., I feel that the things I did in the sessions will help me to make the changes that I want; The different activities we did in the sessions were helpful; The facilitator valued my opinion; The different activities we did in the sessions were helpful). Similar satisfaction questions have been used in prior research [106–108].

### Analyses

Given the planned sample size (i.e., n = 60), sophisticated modeling and adjustments for non-response might not be possible, and the bulk of the analyses will be primarily descriptive. We will estimate the outcomes’ variability in this population and more generally assess the hypothesized trends to determine the intervention promise and identify potential mediators for future studies. Analyses will use the standard intent-to-treat (ITT) approach to examine the effect of offering the MNI intervention to eligible and consented participants. We will attempt to follow up with all participants, regardless of their MNI participation. Our ITT approach will analyze participants as belonging to the group they were randomized to, regardless of their compliance, because excluding those who do not complete the MNI would bias results in favor of MNI, increasing type I errors [109].

We will be able to obtain intervention effect estimates on both the proximal (i.e., efficacy and readiness to change AOD, safe-sex behavior, and networks) and distal outcomes (i.e., AOD use and HIV risk behavior) using a difference in differences (DID) approach [110]. The DID approach is well-suited to the data generated by the adopted study design: for every subject we will have a pre- and post-intervention observation and a randomized group indicator (intervention or control). We will implement the DID approach by fitting a mixed-effects model in which participants are treated as random effects while time, the intervention group indicator, and their interaction are treated as fixed effects. This modeling approach accounts properly for the two repeated measures’ correlation on each participant and tends to produce efficient intervention effect estimates [111]. The intervention effect estimate is given by the coefficient of the interaction between the time and intervention indicators.

## Discussion

This study addresses a critical public health problem: how to address high risk AOD use and HIV risk behaviors in a homeless population transitioning to a stable housing program. The MNI intervention is innovative in that it uses technology to assist individuals to change their own social networks. To our knowledge, this is the first social network intervention that targets formerly homeless individuals as they transition into housing that shares the social network information directly to intervention participants using a MI approach so that participants can act as informed change agents in their social environments. Unlike previous social network interventions that are primarily focused on the diffusion of an innovation throughout a bounded network of individuals sharing a social relationship [24], this intervention will allow individuals to target changes in their own personal networks that span across different types of network contacts and social worlds.

In sum, incorporating network visualizations is an innovative approach to designing an intervention as it incorporates both network structure and composition. Moreover, delivering the social network information electronically utilizing a MI approach will allow for consistent intervention delivery while remaining personally relevant to each participant. The pilot test of this intervention will help to determine if it has promise for expanded tests of its efficacy. We expect that this study will provide the foundation for a larger trial that will address the current study’s limitations with a larger sample, a longer follow-up assessment, controls on unintended network effects, and measures of high-risk behavior and network changes that do not rely on self-reports. The ultimate goal is to provide an easy-to-use and effective tool to help individuals reduce behaviors that negatively impact their health, such as AOD use and high-risk sexual behavior. In addition, although this social network intervention targets HF residents, we believe that many of the innovations developed in this project will provide a template for the development of other social network interventions targeting diverse health outcomes and populations. The software EgoWeb, which has been modified as part of this project, is easy to program, open source and freely available on a code sharing software website (www.github.com/qualintitative/egoweb). This may help reduce the barriers for non-experts in social network analysis to enhance other existing health interventions with a social network component to address some of the social determinants of health outcomes.

## Conclusions

The current study will develop and test a computer-assisted intervention designed to reduce AOD and HIV-related behaviors. This study focuses on developing and testing technology as part of a motivational social network intervention for residents who are transitioning out of homelessness and into HF residency because their networks are likely to be undergoing a period of heightened instability. Results of this pilot test will inform larger clinical trials and has the potential to be used in the future as part of interventions designed to promote behavior change.

## Abbreviations

HF: Housing First
AOD: alcohol or other drug
TF: treatment first
MI: motivational interviewing
PSH: permanent supportive housing
SRHT: Skid Row Housing Trust
SRO: Housing, Single Room Occupancy Housing Corporation
AUDIT-C: Alcohol Use Disorders Identification Test
DAST: Drug Abuse Screen Test
ITT: intent-to-treat
DID: difference in differences
SPC: shelter plus care
CAPI: computer-assisted personal interviewing
RTCQ: Readiness to Change Questionnaire
RTCQ-SB: Readiness to Change Risky Sexual Behavior
AASE: Alcohol Abstinence Self-Efficacy Scale
QFI: Quantity Frequency Index
CSQ-8: Client Satisfaction Questionnaire (CSQ-8)

## References

[1] Wolitski RJ, Kidder DP, Fenton KA (2007). HIV, homelessness, and public health: critical issues and a call for increased action. AIDS Behav.

[2] Fazel S, Khosla V, Doll H, Geddes J (2008). The prevalence of mental disorders among the homeless in western countries: systematic review and meta-regression analysis. PLoS Med.

[3] Allen DM, Lehman JS, Green TA, Lindegren ML, Onorato IM, Forrester W (1994). HIV-infection among homeless adults and runaway youth, United States, 1989–1992. AIDS.

[4] Culhane DP, Gollub E, Kuhn R, Shpaner M (2001). The co-occurrence of AIDS and homelessness: results from the integration of administrative databases for AIDS surveillance and public shelter utilisation in philadelphia. J Epidemiol Community Health.

[5] Booth BM, Sullivan G, Koegel P, Burnam A (2002). Vulnerability factors for homelessness associated with substance dependence in a community sample of homeless adults. Am J Drug Alcohol Abuse.

[6] Rhoades H, Wenzel SL, Golinelli D, Tucker JS, Kennedy DP, Green HD (2011). The social context of homeless men’s substance use. Drug Alcohol Depend.

[7] Tucker JS, Kennedy D, Ryan G, Wenzel SL, Golinelli D, Zazzali J (2009). Homeless women’s personal networks: implications for understanding risk behavior. Hum Organ.

[8] Wenzel SL, Green HD, Tucker JS, Golinelli D, Kennedy DP, Ryan G (2009). The social context of homeless women’s alcohol and drug use. Drug Alcohol Depend.

[9] Davey-Rothwell MA, Latimore A, Hulbert A, Latkin CA (2011). Sexual networks and housing stability. J Urban Health Bull NY Acad Med.

[10] Robertson MJ, Clark RA, Charlebois ED, Tulsky J, Long HL, Bangsberg DR (2004). HIV seroprevalence among homeless and marginally housed adults in San Francisco. Am J Public Health.

[11] Kidder DP, Wolitski RJ, Royal S, Aidala A, Courtenay-Quirk C, Holtgrave DR (2007). Access to housing as a structural intervention for homeless and unstably housed people living with HIV: rationale, methods, and implementation of the housing and health study. AIDS Behav.

[12] Milby JB, Schumacher JE, Wallace D, Vuchinich R, Mennemeyer ST, Kertesz SG (2010). Effects of sustained abstinence among treated substance-abusing homeless persons on housing and employment. Am J Public Health.

[13] Milby JB, Schumacher JE, Wallace D, Freedman MJ, Vuchinich RE (2005). To house or not to house: the effects of providing housing to homeless substance abusers in treatment. Am J Public Health.

[14] Milby JB, Schumacher JE, Vuchinich RE, Freedman MJ, Kertesz S, Wallace D (2008). Toward cost-effective initial care for substance-abusing homeless. J Subst Abuse Treat.

[15] Kertesz SG, Crouch K, Milby JB, Cusimano RE, Schumacher JE (2009). Housing first for homeless persons with active addiction: are we overreaching?. Milbank Q.

[16] Padgett DK (2007). There’s no place like (a) home: ontological security among persons with serious mental illness in the United States. Soc Sci Med.

[17] Padgett DK, Stanhope V, Henwood BF, Stefancic A (2011). Substance use outcomes among homeless clients with serious mental illness: comparing housing first with treatment first programs. Community Ment Health J.

[18] Padgett DK, Gulcur L, Tsemberis S (2006). Housing first services for people who are homeless with co-occurring serious mental illness and substance abuse. Res Soc Work Pract.

[19] Tsemberis S, Kent D, Respress C (2012). Housing stability and recovery among chronically homeless persons with co-occurring disorders in Washington, DC. Am J Public Health.

[20] Larimer ME, Malone DK, Garner MD, Atkins DC, Burlingham B, Lonczak HS (2009). Health care and public service use and costs before and after provision of housing for chronically homeless persons with severe alcohol problems. JAMA.

[21] Gladwell M. Million-dollar Murray: why problems like homelessness may be easier to solve than to manage. The New Yorker; 2006. www.gladwell.com/pdf/murray.pdf. Accessed 7 Feb 2012.

[22] Graves F, Sayfan H. ‘Housing first,’ a radical new approach to ending chronic homelessness, is gaining ground in Boston. Boston Globe; 2007. http://www.boston.com/news/globe/ideas/articles/2007/06/24/first_things_first. Accessed 30 Nov 2011.

[23] Whittaker E, Swift W, Flatau P, Dobbins T, Schollar-Root O, Burns L (2015). A place to call home: study protocol for a longitudinal, mixed methods evaluation of two housing first adaptations in Sydney, Australia. BMC Public Health.

[24] Valente TW (2012). Network interventions. Science.

[25] Valente TW (2010). Social networks and health. Models, methods, and applications.

[26] Fisher JD (1988). Possible effects of reference group-based social influence on AIDS risk behavior and AIDS prevention. Am Psychol.

[27] Latkin C, Mandell W, Oziemkowska M, Celentano D, Vlahov D, Ensminger M (1995). Using social network analysis to study patterns of drug use among urban drug users at high risk for HIV/AIDS. Drug Alcohol Depend.

[28] Smith KP, Christakis NA (2008). Social networks and health. Annu Rev Sociol.

[29] Berkman LF, Glass T, Brissette I, Seeman TE (2000). From social integration to health: Durkheim in the new millennium. Soc Sci Med.

[30] Reitzes DC, Crimmins TJ, Yarbrough J, Parker J (2011). Social support and social network ties among the homeless in a downtown Atlanta park. J Community Psychol.

[31] Stablein T (2011). Helping friends and the homeless milieu: social capital and the utility of street peers. J Contemp Ethnogr.

[32] Wolch JR, Rahimian A, Koegel P (1993). Daily and periodic mobility patterns of the urban homeless. Prof Geogr.

[33] Hawkins RL, Abrams C (2007). Disappearing acts: the social networks of formerly homeless individuals with co-occurring disorders. Soc Sci Med.

[34] Irwin J, LaGory M, Ritchey F, Fitzpatrick K (2008). Social assets and mental distress among the homeless: exploring the roles of social support and other forms of social capital on depression. Soc Sci Med.

[35] Padgett DK, Henwood B, Abrams C, Drake RE (2008). Social relationships among persons who have experienced serious mental illness, substance abuse, and homelessness: implications for recovery. Am J Orthopsychiatry.

[36] Hwang SW, Kirst MJ, Chiu S, Tolomiczenko G, Kiss A, Cowan L (2009). Multidimensional social support and the health of homeless individuals. J Urban Health Bull NY Acad Med.

[37] Hwang SW, Tolomiczenko G, Kouyoumdjian FG, Garner RE (2005). Interventions to improve the health of the homeless—a systematic review. Am J Prev Med.

[38] Zerger S, Strehlow AJ, Gundlapalli AV (2008). Homeless young adults and behavioral health: an overview. Am Behav Sci.

[39] Johnson TP, Fendrich M (2007). Homelessness and drug use—evidence from a community sample. Am J Prev Med.

[40] Brown RA, Kennedy DP, Tucker JS, Golinelli D, Wenzel SL (2013). Monogamy on the street: a mixed methods study of homeless men. J Mixed Methods Res.

[41] Kennedy DP, Tucker JS, Green HD, Golinelli D, Ewing BA (2012). Unprotected sex of homeless youth: results from a multilevel analysis of individual, social network, and relationship factors. AIDS Behav.

[42] Brown RA, Kennedy DP, Tucker JS, Wenzel S, Golinelli D, Wertheimer S (2012). Sex and relationships on the street: how homeless men judge partner risk on Skid Row. AIDS Behav.

[43] Kennedy DP, Wenzel SL, Tucker JS, Green HD, Golinelli D, Ryan GW (2010). Unprotected sex of homeless women living in Los Angeles county: an investigation of the multiple levels of risk. AIDS Behav.

[44] Green HD, Tucker JS, Golinelli D, Wenzel SL (2013). Social networks, time homeless, and social support: a study of men on Skid Row. Netw Sci.

[45] Kennedy DP, Green HD, McCarty C, Tucker JS (2011). Nonexperts’ recognition of structure in personal network data. Field Methods.

[46] Green HD, Tucker JS, Wenzel SL, Golinelli D, Kennedy DP, Ryan GW, Zhou AJ (2012). Association of childhood abuse with homeless women’s social networks. Child Abus Negl.

[47] Valente TW, Ritt-Olson A, Stacy A, Unger JB, Okamoto J, Sussman S (2007). Peer acceleration: effects of a social network tailored substance abuse prevention program among high-risk adolescents. Addiction.

[48] Valente TW, Hoffman BR, Ritt-Olson A, Lichtman K, Johnson CA (2003). Effects of a social-network method for group assignment strategies on peer-led tobacco prevention programs in schools. Am J Public Health.

[49] Centola D (2010). The spread of behavior in an online social network experiment. Science.

[50] Copello A, Orford J, Hodgson R, Tober G, Barrett C, Team UR (2002). Social behaviour and network therapy—basic principles and early experiences. Addict Behav.

[51] Bond J, Kaskutas LA, Weisner C (2003). The persistent influence of social networks and alcoholics anonymous on abstinence. J Stud Alcohol.

[52] Litt MD, Kadden RM, Kabela-Cormier E, Petry N (2007). Changing network support for drinking: initial findings from the network support project. J Consult Clin Psychol.

[53] Litt MD, Kadden RM, Kabela-Cormier E, Petry NM (2009). Changing network support for drinking: network support project 2-year follow-up. J Consult Clin Psychol.

[54] Groh DR, Jason LA, Keys CB (2008). Social network variables in alcoholics anonymous: a literature review. Clin Psychol Rev.

[55] Kelly JF, Stout RL, Magill M, Tonigan JS (2011). The role of alcoholics anonymous in mobilizing adaptive social network changes: a prospective lagged mediational analysis. Drug Alcohol Depend.

[56] Kaskutas LA, Bond J, Humphreys K (2002). Social networks as mediators of the effect of alcoholics anonymous. Addiction.

[57] Mason M, Pate P, Drapkin M, Sozinho K (2011). Motivational interviewing integrated with social network counseling for female adolescents: a randomized pilot study in urban primary care. J Subst Abuse Treat.

[58] McCarty C, Molina JL, Aguilar C, Rota L (2007). A comparison of social network mapping and personal network visualization. Field Methods.

[59] Hogan B, Carrasco JA, Wellman B (2007). Visualizing personal networks: working with participant-aided sociograms. Field Methods.

[60] Luis Molina J, Maya-Jariego Luis Molina, McCarty C, Dominguez S, Hollstein B (2014). Giving meaning to social networks: methodology for conducting and analyzing interviews based on personal network visualizations. Mixed methods social networks research: design and applications.

[61] Rempel GR, Neufeld A, Kushner KE (2007). Interactive use of genograms and ecomaps in family caregiving research. J Fam Nurs.

[62] Tracy EM, Whittaker JK (1990). The social network map—assessing social support in clinical-practice. Fam Soc J Contemp Hum Serv.

[63] Yoon SA (2011). Using social network graphs as visualization tools to influence peer selection decision-making strategies to access information about complex socioscientific issues. J Learn Sci.

[64] Miller WR, Rose GS (2009). Toward a theory of motivational interviewing. Am Psychol.

[65] Hettema J, Steele J, Miller WR (2005). Motivational interviewing. Annu Rev Clin Psychol.

[66] Bem DJ, Berkowitz L (1972). Self-perception theory. Advances in experimental social psychology.

[67] Miller WR, Rollnick S (2002). Motivational interviewing: preparing people for change.

[68] Marsch LA, Grabinski MJ, Bickel WK, Desrosiers A, Guarino H, Muehlbach B (2011). Computer-assisted HIV prevention for youth with substance use disorders. Subst Use Misuse.

[69] Rounsaville BJ, Carroll KM, Onken LS (2001). A stage model of behavioral therapies research: getting started and moving on from stage I. Clin Psychol Sci Pract.

[70] United States Interagency Council on Homelessness. Implementing housing first in permanent supportive housing. 2014. http://usich.gov/resources/uploads/asset_library/Implementing_Housing_First_in_Permanent_Supportive_Housing.pdf. Accessed 7 Aug 2015.

[71] Katzman R, Brown T, Fuld P, Peck A, Schechter R, Schimmel H (1983). Validation of a short orientation–memory–concentration test of cognitive impairment. Am J Psychiatry.

[72] Bradley KA, McDonell MB, Kivlahan DR, Diehr P, Fihn SD (1998). The AUDIT alcohol consumption questions: reliability, validity and responsiveness to change in older male primary care patients. Alcohol Clin Exp Res.

[73] Cocco KM, Carey KB (1998). Psychometric properties of the drug abuse screening test in psychiatric outpatients. Psychol Assess.

[74] Maisto SA, Carey MP, Carey KB, Gordon CM, Gleason JR (2000). Use of the AUDIT and the DAST-10 to identify alcohol and drug use disorders among adults with a severe and persistent mental illness. Psychol Assess.

[75] Skinner HA (1982). The drug abuse screening test. Addict Behav.

[76] de la Haye K, Green HD, Kennedy DP, Zhou A, Golinelli D, Wenzel SL (2012). Who is supporting homeless youth? Predictors of support in personal networks. J Res Adolesc.

[77] Kennedy DP, Wenzel SL, Brown R, Tucker JS, Golinelli D (2013). Unprotected sex among heterosexually active homeless men: results from a multi-level dyadic analysis. AIDS Behav.

[78] D’Amico EJ, Miles JNV, Stern SA, Meredith LS (2008). Brief motivational interviewing for teens at risk of substance use consequences: a randomized pilot study in a primary care clinic. J Subst Abuse Treat.

[79] D’Amico EJ (2005). Brief substance use intervention for primary care.

[80] Osilla KC, D’Amico EJ, Díaz-Fuentes CM, Lara M, Watkins KE (2012). Multicultural web-based motivational interviewing for clients with a first-time DUI offense. Cult Divers Ethn Minor Psychol.

[81] Hogue A, Dauber S, Morgenstern J (2010). Validation of a contemplation ladder in an adult substance use disorder sample. Psychol Addict Behav.

[82] Bluthenthal RN, Gogineni A, Longshore D, Stein M (2001). Factors associated with readiness to change drug use among needle-exchange users. Drug Alcohol Depend.

[83] LaBrie JW, Quinlan T, Schiffman JE, Earleywine ME (2005). Performance of alcohol and safer sex change rulers compared with Readiness To Change Questionnaires. Psychol Addict Behav.

[84] Young AS, Niv N, Chinman M, Dixon L, Eisen SV, Fischer EP (2011). Routine outcomes monitoring to support improving care for schizophrenia: report from the VA Mental Health QUERI. Community Ment Health J.

[85] Donovan DM, Bigelow GE, Brigham GS, Carroll KM, Cohen AJ, Gardin JG (2011). Primary outcome indices in illicit drug dependence treatment research: systematic approach to selection and measurement of drug use end-points in clinical trials. Addiction.

[86] Biener L, Abrams DB (1991). The contemplation ladder: validation of a measure of readiness to consider smoking cessation. Health Psychol.

[87] Steinberg ML, Ziedonis DM, Krejci JA, Brandon TH (2004). Motivational interviewing with personalized feedback: a brief intervention for motivating smokers with schizophrenia to seek treatment for tobacco dependence. J Consult Clin Psychol.

[88] Heather N, Rollnick S, Bell A (1993). Predictive validity of the Readiness to Change Questionnaire. Addiction.

[89] Heather N, Gold R, Rollnick S (1991). Readiness to change questionnaire: user’s manual.

[90] Rollnick S, Heather N, Gold R, Hall W (1992). Development of a short readiness to change questionnaire for use in brief, opportunistic interventions among excessive drinkers. Br J Addict.

[91] DiClemente CC, Carbonari JP, Montgomery RP, Hughes SO (1994). The alcohol abstinence self-efficacy scale. J Stud Alcohol Drugs.

[92] Marin BV, Tschann JM, Gomez CA, Gregorich S (1998). Self-efficacy to use condoms in unmarried Latino adults. Am J Community Psychol.

[93] Kennedy DP, Wenzel SL, Brown R, Tucker JS, Golinelli D (2013). Unprotected sex among heterosexually active homeless men: results from a multi-level dyadic analysis. AIDS Behav.

[94] Tucker JS, Wenzel SL, Golinelli D, Ryan G, Zhou A, Beckman R (2010). Is substance use a barrier to protected sex among homeless women? Results from between- and within-subjects event analyses. J Stud Alcohol Drugs.

[95] Wenzel SL, Tucker JS, Golinelli D, Green HD, Zhou A (2010). Personal network correlates of alcohol, cigarette, and marijuana use among homeless youth. Drug Alcohol Depend.

[96] Wenzel S, Holloway I, Golinelli D, Ewing B, Bowman R, Tucker J. Social networks of homeless youth in emerging adulthood. J Youth Adolesc. 2011. doi:10.1007/s10964-011-9709-8. 10.1007/s10964-011-9709-8 PMC3227762 21863378

[97] Golinelli D, Ryan G, Green HD, Kennedy DP, Tucker JS, Wenzel SL (2010). Sampling to reduce respondent burden in personal network studies and its effect on estimates of structural measures. Field Methods.

[98] McCarty C, Killworth PD, Rennell J (2007). Impact of methods for reducing respondent burden on personal network structural measures. Soc Netw.

[99] Longabaugh R, Wirtz PW, Zywiak WH, O’Malley SS (2010). Network support as a prognostic indicator of drinking outcomes: the COMBINE Study. J Stud Alcohol Drugs.

[100] Zywiak WH, Neighbors CJ, Martin RA, Johnson JE, Eaton CA, Rohsenow DJ (2009). The important people drug and alcohol interview: psychometric properties, predictive validity, and implications for treatment. J Subst Abuse Treat.

[101] Papp LM, Witt NL (2010). Romantic partners’ individual coping strategies and dyadic coping: implications for relationship functioning. J Fam Psychol.

[102] Thomas OH (1997). Measuring excessive alcohol use in college drinking contexts: the Drinking Context Scale. Addict Behav.

[103] Kennedy DP, Brown R, Golinelli D, Wenzel S, Tucker JS, Wertheimer S. Masculinity and HIV risk among homeless men in Los Angeles. Psychol Men Masc. 2012. doi:10.1037/a0027570. 10.1037/a0027570 PMC3667623 23730216

[104] McCarty C. Measuring structure in personal networks. J Soc Struct. 2002.

[105] Wasserman S, Faust K (1994). Social network analysis: methods and applications.

[106] Marlatt GA, Baer JS, Kivlahan DR, Dimeff LA, Larimer ME, Quigley LA (1998). Screening and brief intervention for high-risk college student drinkers: results from a 2-year follow-up assessment. J Consult Clin Psychol.

[107] D’Amico EJ, Osilla KC, Hunter SB (2010). Developing a group motivational interviewing intervention for adolescents at-risk for developing an alcohol or drug use disorder. Alcohol Treat Q.

[108] Osilla KC, Zellmer SP, Larimer ME, Neighbors C, Marlatt GA (2008). A brief intervention for at-risk drinking in an employee assistance program. J Stud Alcohol Drugs.

[109] Hewitt CE, Torgerson DJ, Miles JNV (2006). Is there another way to take account of noncompliance in randomised trials?. Can Med Assoc J.

[110] Donald SG, Lang K (2007). Inference with difference-in-differences and other panel data. Rev Econ Stat.

[111] Yang L, Tsiatis AA (2001). Efficiency study of estimators for a treatment effect in a pretest-posttest trial. Am Stat.

